# Evaluating macro‐ and micronutrients and food groups intake with the risk of developing inflammatory bowel disease: Is there any association?

**DOI:** 10.1002/fsn3.2988

**Published:** 2022-07-26

**Authors:** Farnaz Farsi, Negin Tahvilian, Azadeh Heydarian, Sara Karimi, Sara Ebrahimi, Nasser Ebrahimi‐Daryani, Sanam Tabataba‐Vakili, Javad Heshmati, Marjan Mokhtare

**Affiliations:** ^1^ Minimally Invasive Surgery Research Center Iran University of Medical Sciences Tehran Iran; ^2^ Nutrition and Food Security Research Center Shahid Sadoughi University of Medical Sciences Yazd Iran; ^3^ Department of Nutrition, School of Public Health Shahid Sadoughi University of Medical Sciences Yazd Iran; ^4^ Student Research Committee, Department of Nutrition, School of Public Health Iran University of medical sciences Tehran Iran; ^5^ Department of Clinical Nutrition and Dietetics, Faculty of Nutrition and Food Technology, National Nutrition and Food Technology Research Institute, Shahid Beheshti University of Medical Sciences Tehran Iran; ^6^ The Ritchie Centre Hudson Institute of Medical Research, Monash Medical Centre, Clayton Melbourne VIC Australia; ^7^ Division of Gastroenterology, Imam Khomeini Hospital, School of Medicine Tehran University of Medical Sciences Tehran Iran; ^8^ Department of Medicine Shahid Beheshti University of Medical Sciences Tehran Iran; ^9^ Department of Nutritional Science, School of Nutritional Science and Food Technology Kermanshah University of Medical Sciences Kermanshah Iran; ^10^ Rasoul Akram Hospital Clinical Research Development Center Iran University of Medical Sciences Tehran Iran

**Keywords:** Crohn disease, diet, inflammatory bowel disease, nutrient, ulcerative colitis

## Abstract

Growing clinical evidence represented that certain dietary components are involved in inflammatory bowel disease (IBD) development and progression. This research, therefore, aimed to evaluate whether there exists any relationship between nutrients and IBD. This case–control study from 2017 to 2019 was performed on 145 newly diagnosed IBD patients and 145 BMI‐, sex‐, and age‐matched healthy controls who were recruited from a hospital clinic. A validated 168‐item food frequency questionnaire was completed by each participant. Anthropometric measurements and physical activity levels were measured for all participants. Stata software was used to analyze all data. Of the 234 study individuals who participated, 112 were IBD patients and 122 were healthy people. The higher amount of seafood and cholesterol was related to an increased risk of IBD and ulcerative colitis development; however, individuals who had a higher intake of calcium were less likely to have Crohn's compared to the healthy group. There was a positive relation between honey and jam, seafood, organ meats, salt, fruits on trees, fruit juice, olives, and nuts and the probability of IBD, but there was a negative association between refined grains, potatoes, salty snacks, legumes, dairy, and cruciferous and the probability of IBD. Higher consumption of seafood and cholesterol was positively connected with a higher risk of IBD development in the current case–control study. A substantial association was seen between honey and jam, seafood, organmeats, salt, fruit on trees, fruit juice, olives, and nut consumption and IBD developement.

## Graphical abstract

This study aimed to evaluate whether there exists any relationship between nutrients and IBD. Main finding showed that higher consumptions of seafood and cholesterol was positively connected with a higher risk of IBD development.

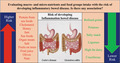

## 1 INTRODUCTION

Inflammatory bowel disease (IBD), a chronic relapsing inflammatory disease of the digestive tract, is characterized by predominantly ulcerative colitis (UC) and Crohn's disease (CD), which differ in pathology and clinical features (Mirmiran, Moslehi, et al., 2019; Ye et al., 2015). Its clinical manifestations contain abdominal pain, fatigue, weight loss, diarrhea, urge, rectal bleeding, fever, growth disturbances, and puberty latency (Lindberg et al., 2000). According to recent reports, both the incidence and prevalence of IBD among the general population are rapidly rising all over the world (Brooks et al., 2016; Malekzadeh et al., 2016). Despite the dramatic increase in the incidence of IBD in Asia over the past decade, there is no exact estimate of this disease in Iran (Malekzadeh et al., 2016; Ng et al., 2016; Sherkat et al., 2018). It seems that IBD can become one of the considerable challenges to health in the near future (Malekzadeh et al., 2016). Due to the illness onset in early adulthood and its chronic nature, IBD could lead to a remarkable decline in the quality of life and could impose a heavy burden on the health care system at the same time (Barbalho et al., 2016; Farsi et al., 2021; Malekzadeh et al., 2016).

IBD is a multifactorial disease, and the main pathogenesis factors consist of host genetic predisposition or background and environmental factors such as tobacco use, diet, antibiotic therapy, vitamin D deficiency, food freezing, socioeconomic status, stress, appendectomy, and westernized lifestyle, although the exact causes and pathogenesis of this disease are still not fully recognized (Burke et al., 2017; Jakobsen et al., 2013).

Both dietary habits and ingredients of diet could also affect the variety of intestinal microbiota and host immunity, triggering intestinal inflammation or gut permeability(Mirmiran, Moslehi, et al., 2019; Rashvand et al., 2015). Recent studies on dietary intake and developing IBD illustrated that nutrients (fats, carbohydrates, and protein) are able to modify the physiology of the digestive system through regulating gut hormone release and gut microenvironment by influencing gut microbiota function and composition. For this reason, several case–control studies have been conducted to assess the relation between preillness intake of nutrients and the development and progression of IBD, which have shown controversial results (Bischoff et al., 2020; Rizzello et al., 2019).

In a systematic review of 19 clinical studies, Hou et al. revealed that the risk of developing UC and CD elevated among individuals who had a higher amount of PUFAs, *n*‐6 fatty acids, and meats; meanwhile, high intake of dietary fiber and fruits could reduce risk of CD, but not UC (Hou et al., 2011). Besides, the studies in North America and Western Europe indicated that there is a positive association between IBD incidence and higher intake of processed foods, such as sugars, and lower intake of fruits and vegetables (Dworzanski et al., 2016). The existing evidence suggested no relationship between intake of total fat, saturated fats, unsaturated fats, *n*‐6, and *n*‐3 PUFA with the risk of CD or UC (Bischoff et al., 2020). A case–control study also displayed an increased risk of UC when consuming more processed meat, red meat, and organ meat, but the stuffiest intake of dietary vitamin C and folate was related to a lower risk of UC. However, there still are controversial data regarding the exact role of diet in the onset and progression of IBD (Rizzello et al., 2019).

To our knowledge, no case–control study has evaluated relationships between total micro‐ and macronutrient and food group intake with the risk of IBD development in the population. As a result, the aim of this study was to examine the association between total micro‐/macronutrient and food group intake in developing IBD.

## 2 MATERIALS AND METHODS

### 2.1 Study design

The current study was conducted as a case–control study from 2017 to 2019 on newly diagnosed IBD patients (as a case group) and healthy age‐, body mass index (BMI)‐, and sex‐matched individuals (as a control group). All eligible participants, who were referred to the gastroenterology clinic and/or ward of Rasoul‐e‐Akram Hospital, Tehran, Iran, were enrolled in this study. The study protocol was confirmed at the Ethics Committee of Iran University of medical sciences (IUMS), Tehran, Iran, with the Ethics number of IR. IUMS.REC139631962. All the conditions of performing the study were explained in detail to participants and an informed written consent also was taken from each participant.

### 2.2 Study participants

This case–control study was performed on 290 participants who fulfilled the inclusion criteria. Overall, 145 new cases of patients with IBD, who have been diagnosed by a gastroenterologist based on clinical, biomarker, colonoscopy, and histopathologic findings, and 145 age‐, BMI‐, and sex‐matched healthy controls were enrolled in the study. Eligibility criteria were as follows: their tendency to take part in the study, men and women older than 18 years, body mass index (BMI) higher than 18.5 or lower than 30 kg/m^2^, and having daily activity. The exclusion criteria included: a history of systemic and/or metabolic diseases that may perceivably be linked to dietary intake (such as hypertension, hypothyroidism, diabetes mellitus, cardiovascular disease, gout, and hyperlipidemia), history of abdominal surgery, celiac disease, scleroderma, bile acid‐related diarrhea, pancreatic insufficiency, food allergy, infection with cytomegalovirus, clostridium difficile and tuberculosis, any history of probiotics, omega‐3 fatty acids and fish oil consumption, pregnancy, breastfeeding, athletes, bedridden person, noncooperative patients, and any hospitalization for a flare up disease within the past 6 months prior to the study.

### 2.3 Measurements

In the first stage, all information of each participant including age, past drug history, socioeconomic/education status, alcohol consumption, smoking history, and IBD subtypes were collected through the completion of the general information questionnaire. What is more, a valid and reliable food frequency questionnaire (FFQ) of 168 items was applied to evaluate the usual dietary intakes of study individuals over the past year through private face‐to‐face interview, which was carried out by trained researchers for each participant personally (Rezazadeh et al., 2020). According to the amounts ingested on a daily, weekly, or monthly basis, the consumed food items in the FFQ were converted into grams using United States Department of Agriculture (USDA) portion sizes and household measures. N4 software was also used to calculate the average amounts of food, energy, and nutrients consumed. A total of 36 food groups were created based on the similarity of the nutrient composition of foods (Cheraghi et al., 2018; Esmaillzadeh & Azadbakht, 2008; Mirmiran, Ziadlou, et al., 2019). A list of the food groups is provided in Table S1.

For anthropometric measurements, standing height was recorded using a nonstretch tape meter mounted on a wall with 0.5 cm accuracy without shoes, and heels sticking to the wall in a normal position. Body weight was measured for subjects standing without shoes using a calibrated digital scale (Seca 644 hand‐rail scale, Seca Corp, Hanover, Maryland) with an accuracy of 100 grams. Body mass index (BMI) was then calculated as the ratio of an individual's weight in kilograms divided by square of person's height in meters (kg/m^2^). To assess patients' physical activity status, the *International Physical Activity Questionnaires Short Form* (IPAQ‐SF) of self‐report questions for each participant was completed. The IPAQ data were calculated according to MET scores (MET‐hour/week) by multiplying the number of hours and days of that activity (Moghaddam et al., 2012).

### 2.4 Statistical analysis

Stata (version 16.0; Stata Corp) was used to perform the data analysis. A *p*‐value of <.05 was considered statistically significant. The number and percentage of participants were reported for categorical variables and means and standard errors were reported for continuous variables. Multinominal logistic regression analysis and logistic regression analysis were used to examine associations among IBD categories (categorical, dependent and binary, dependent, respectively), sociodemographic characteristics (categorical and independent), nutrient intakes (continuous and independent), and food groups intake (continuous and independent). The analysis was adjusted for confounders including age, sex, education, status of marriage and total energy, smoking, BMI, and physical activity. A complete case analysis approach was used to manage missing data for all variables.

## 3 RESULTS

**FIGURE 1:**
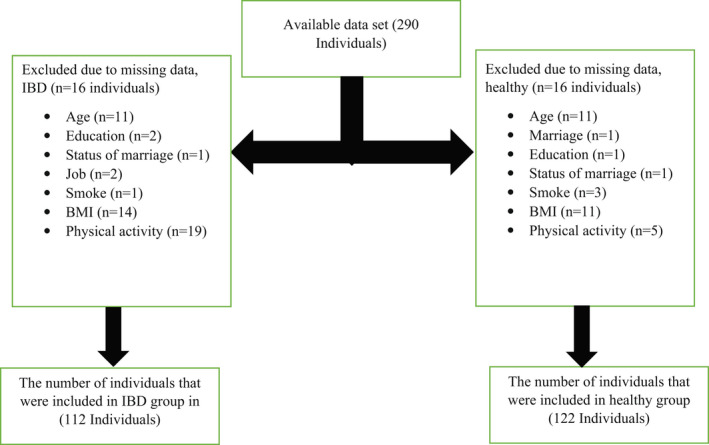
Participant flow diagram

Of the 290 individuals who participated in this study, 56 individuals were excluded due to missing data, and a total of 234 individuals were included in the present analysis (Figure 1). The majority of individuals in healthy and UC were males and married (60% and 63%, respectively); meanwhile, the majority of participants with CD were single. The majority of participants in all groups had the highest level of education and were unemployed, housekeepers, students, and retired. The sociodemographic characteristics of participants were not among between UC, CD, and healthy participants. The sociodemographic characteristics of participants (healthy, UC, and CD groups) are presented in Table 1. While the intake of total protein from seafoods and cholesterol is higher in UC, calcium intake is less in CD compared to healthy participants. The total mean nutrient intake is presented in Table 2. According to the obtained results, participants with a higher intake of proteins from seafoods were more probable to have UC, while patients who had a higher intake of calcium were less likely to have CD compared to healthy individuals. Participants with higher intake of cholesterol were more likely to have UC. The association between the macro‐and micro‐nutrients and the relative risk of developing IBD subgroups (UC and CD groups) is shown in Table 3. There was a positive relationship between protein from seafoods and cholesterol and the risk of developing IBD. The relationship between nutrient intake and the risk of IBD development versus the control group is demonstrated in Table 4. Participants who had a higher intake of whole grains, honey and jam, seafoods, organ meats, green vegetables, onions, salt, fruits on trees, fruit juice, olives, and nuts were more likely to have UC; whereas the higher intake of refined grains, potatoes, legumes, high‐fat dairy products, and cruciferous was less likely to have UC in comparison to healthy subjects. Additionally, individuals who had a higher intake of organ meats, fruits on trees, hydrogenated fats, and olives were more likely to have CD, while participants with higher intake of refined grains, sweets, salty snacks, legumes, and high‐fat dairy products were less probable to have CD. The relationship between food groups and the relative risk of developing IBD categories (UC and CD groups) is displayed in Table 5. There was a positive relation between honey and jam, seafoods, organ meats, salt, fruits on trees, fruit juice, olives, and nuts and the probability of IBD; meanwhile, there was a negative association between refined grains, potatoes, salty snacks, legumes, high‐fat dairy, and cruciferous and the probability of IBD. The association between food groups and the risk of developing IBD in comparison to healthy individuals is illustrated in Table 6.

**TABLE 1:** The sociodemographic characteristics of participants by healthy and IBD categories (*N* = 234)

Characteristics	Healthy (*n* = 122)	UC (*n* = 86)	*p* value^a^	CD (*n* = 26)	*p* value^b^
Age	36.4 (11.6)	40.8 (12.7)	.313	31.6 (10.8)	.056
Sex			.860		.462
Males	49 (40.2)	32 (37.2)	—	13 (50)	
Females	73 (59.8)	54 (62.8)	—	13 (50)	
Education level^c^			.627		.277
Low	3 (2.5)	7 (8.1)	—	1 (3.9)	—
Medium	32 (26.2)	27 (31.4)	—	6 (23.1)	—
High	87 (71.3)	52 (60.5)	—	19 (73.1)	—
Marriage status			.322		.919
Single	55 (45.1)	29 (33.7)	—	16 (61.5)	—
Married	67 (54.9)	57 (66.3)	—	10 (38.5)	—
Job^d^			.182		.732
Group 1	51 (41.8)	45 (52.3)	—	14 (53.9)	—
Group 2	29 (23.8)	20 (23.3)	—	1 (3.9)	—
Group 3	42 (34.4)	21 (24.4)	—	11 (42.3)	—
Smoke			.267		.542
Nonsmokers	98 (81.7)	75 (87.2)	—	21 (80.8)	—
Smokers	22 (18.3)	11 (12.8)	—	5 (19.2)	—
BMI	23.3 (3.3)	24.1 (3.8)	.256	23.7 (4.5)	.190
Physical activity (MET‐min/week)	841 (1419)	1208 (1358)	.060	1788 (3904)	.051

Abbreviations: BMI, body mass index (kg/m^2^); CD, Crohn's disease; UC, ulcerative colitis.

^a^ Wald test was used in multinomial logistic regression analysis for the association between characteristics and colitis ulcerative; the healthy group was considered as the reference group.

^b^ Wald test was used in multinomial logistic regression analysis for the association between characteristics and Crohn's disease; the healthy group was considered as the reference group. Data are presented as mean (SD) or *n* (%).

^c^ Low (no formal schooling, less than primary school, and the primary school completed), medium (secondary school completed and high school completed), and high (college/university completed).

^d^ Group 1 (unemployed, housekeepers, students, and retired), Group 2 (self‐employed such as laborers and businessmen), and Group 3 (employed such as teachers and office workers).

**TABLE 2:** The mean and standard deviation of macronutrients and some micronutrients intake over IBD categories (*N* = 234)

Nutrient intakes	Healthy (*n* = 122)	UC (*n* = 86)	CD (*n* = 26)
Total energy (kcal/day)	2070 (676)	2150 (673)	1981 (387)
Total CHO (g/day)	323 (122)	334 (109)	300 (56)
Total protein (g)	81 (31)	82 (31)	78 (22)
Total protein from red meat (g/day)	6.1 (6.6)	6.8 (4.5)	7.3 (5.4)
Total protein from white meat (g/day)	2.9 (1.8)	3 (1.8)	3.4 (2.5)
Total protein from seafoods (g/day)	2.3 (3.2)	4.5 (4.1)	3.7 (5.3)
Total protein from processed meats (g/day)	0.69 (1.1)	0.45 (0.75)	0.52 (0.76)
Total fat (g/day)	62 (26)	66 (26)	53 (22)
Cholesterol (mg/day)	188 (101.8)	225 (118.4)	240 (117.6)
SFAs (mg/day)	20 (8.9)	21.2 (9.4)	19.9 (7.5)
PUFAs (mg/day)	13.5 (6.1)	13.2 (5.7)	13 (5.6)
Iron (mg/day)	16 (5.8)	17 (6.3)	15 (3.8)
Calcium (mg/day)	942 (412)	914 (354)	796 (266)
Folate (mcg/day)	499 (146)	481 (155)	464 (123)
TFAs (mg/day)	2.3 (2.5)	1.9 (2.8)	2.4 (3.9)
Total fiber (g/day)	39.5 (17.9)	37.8 (16.9)	34.3 (14.8)

*Note*: Data are presented as mean (SD).

Abbreviations: CD, Crohn's disease; CHO, carbohydrate; g, gram; kcal, kilocalorie; mcg, microgram; mg, milligram; PUFAs, polyunsaturated fatty acids; SFAs, saturated fatty acids; TFAs, trans‐fatty acids; UC, ulcerative colitis.

**TABLE 3:** Multinomial logistic regression analysis between IBD categories and nutrient intakes (N = 234)

Nutrient intakes	UC (*n* = 86)			CD (*n* = 26)		
	RR ratio	95% CI	*p* value^a^	RR ratio	95% CI	*p* value^a^
Total energy (kcal/day)	1.00	0.99, 1.00	.440	1.00	0.99, 1.00	.125
Total CHO (g/day)	1.00	0.99, 1.00	.527	1.00	0.99, 1.00	.073
Total protein (g)	1.00	0.99, 1.01	.765	0.99	0.97, 1.00	.110
Total protein from red meat (g/day)	1.02	0.97, 1.08	.410	1.00	0.93, 1.08	.950
Total protein from white meat (g/day)	1.02	0.87, 1.19	.839	1.11	0.89, 1.38	.371
Total protein from seafoods (g/day)	1.19	1.07, 1.32	.001	1.13	0.99, 1.28	.074
Total protein from processed meats (g/day)	0.78	0.51, 1.17	.228	0.55	0.29, 1.06	.075
Total fat (g/day)	1.01	0.99, 1.02	.344	0.99	0.97, 1.01	.450
Cholesterol (mg/day)	1.00	1.00, 1.01	.031	1.00	0.99, 1.01	.098
SFAs (mg/day)	1.02	0.98, 1.05	.312	0.98	0.93, 1.04	.511
PUFAs (mg/day)	0.99	0.94, 1.04	.596	0.93	0.85, 1.02	.114
Iron (mg/day)	1.01	0.96, 1.07	.570	0.92	0.84, 1.01	.071
Calcium (mg/day)	0.99	0.99, 1.00	.477	0.99	0.99, 1.00	.048
Folate (mcg/day)	0.99	0.99, 1.00	.306	0.99	0.99, 1.00	.063
TFAs (mg/day)	0.95	0.85, 1.05	.310	1.02	0.88, 1.19	.795
Total fiber (g/day)	0.99	0.98, 1.01	.409	0.98	0.95, 1.00	.099

Abbreviations: CD, Crohn's disease; CHO, carbohydrate; g, gram; IBD, inflammatory bowel diseases; kcal, kilocalorie; mcg, microgram; mg, milligram; PUFAs, polyunsaturated fatty acids; RR, relative risk; SFAs, saturated fatty acids; TFAs, trans‐fatty acids; UC, ulcerative colitis.

^a^ Wald test is used in multinomial logistic regression analysis for the association between nutrient intakes (continuous and independent) and IBD categories (categorical and dependent), adjusted for age, sex, education, job, marriage, smoke, BMI, and physical activity; the healthy group was considered as a reference group (relative risk ratio 1.00).

**TABLE 4:** Logistic regression analysis between nutrient intake and IBD (*N* = 234)

Nutrient intakes	IBD^a^		
	Odds ratio	95% CI	*p* value^b^
Total energy (kcal/day)	1.00	0.99, 1.00	.950
Total CHO (g/day)	0.99	0.99, 1.00	.872
Total protein (g)	0.99	0.99, 1.01	.737
Total protein from red meat (g/day)	1.02	0.97, 1.07	.486
Total protein from white meat (g/day)	1.04	0.90, 1.20	.595
Total protein from seafoods (g/day)	1.18	1.06, 1.30	.002
Total protein from processed meats (g/day)	0.72	0.49, 1.05	.087
Total fat (g/day)	1.00	0.99, 1.01	.599
Cholesterol (mg/day)	1.00	1.00, 1.01	.019
SFAs (mg/day)	1.01	0.98, 1.04	.541
PUFAs (mg/day)	0.97	0.93, 1.02	.305
Iron (mg/day)	0.99	0.95, 1.04	.810
Calcium (mg/day)	0.99	0.99, 1.00	.173
Folate (mcg/day)	0.99	0.99, 1.00	.117
TFAs (mg/day)	0.96	0.87, 1.06	.421
Total fiber (g/day)	0.99	0.97, 1.01	.176

Abbreviations: CHO, carbohydrate; g, gram; IBD, inflammatory bowel diseases; kcal, kilocalorie; mcg, microgram; mg, milligram; PUFAs, polyunsaturated fatty acids; SFAs, saturated fatty acids; TFAs, trans‐fatty acids.

^a^ IBD (ulcerative colitis and Crohn's disease).

^b^ Wald test in logistic regression analysis for the association between nutrient intakes (continuous and independent) and IBD (binary and dependent), and adjusted for age, sex, education, job, marriage, smoke, BMI, and physical activity; the healthy group was considered as a reference group (odds ratio 1.00).

**TABLE 5:** Multinominal logistic regression analysis between IBD categories and food groups intake (*N* = 234)

Food groups intake	UC (*n* = 86)			CD (*n* = 26)		
	RR ratio	95% CI	*p*‐value^a^	RR ratio	95% CI	*p*‐value^a^
Whole grains	1.00	1.00, 1.01	.006	0.99	0.99, 1.00	.236
Refined grains	0.996	0.992, 0.998	.002	0.99	0.992, 0.999	.041
Potato	0.96	0.95, 0.98	<.001	0.97	0.94, 0.99	.10
Sweets	0.99	0.98, 1.01	.298	0.95	0.92, 0.99	.013
Sugars	0.99	0.96, 1.01	.223	0.98	0.95, 1.02	.373
Honey and jam	1.06	1.01, 1.11	.020	1.03	0.97, 1.10	.332
Salty snacks	0.98	0.96, 1.01	.129	0.95	0.91, 0.99	.038
Soft drinks	0.99	0.99, 1.00	.345	0.99	0.99, 1.00	.414
Tea	1.00	0.99, 1.00	.841	1.00	0.99, 1.00	.356
Coffee	1.00	0.99, 1.01	.072	1.00	0.99, 1.01	.825
Legumes	0.90	0.85, 0.95	<.001	0.89	0.82, 0.97	.006
Red meat	0.99	0.98, 1.01	.538	0.99	0.97, 1.01	.383
Poultry meat	1.00	0.96, 1.05	.839	1.03	0.97, 1.10	.371
Seafood	1.04	1.02, 1.07	.001	1.03	0.99, 1.06	.072
Processed meat	0.97	0.91, 1.02	.237	0.92	0.84, 1.01	.075
Organ meat	1.10	1.04, 1.16	.001	1.10	1.02, 1.16	.007
Eggs	1.00	0.99, 1.02	.704	1.01	0.99, 1.03	.193
Fast foods	0.97	0.94, 1.01	.109	0.99	0.95, 1.02	.464
Low‐fat dairy	0.99	0.99, 1.00	.895	0.99	0.99, 1.00	.255
High‐fat dairy	0.98	0.98, 0.99	<.001	0.98	0.96, 0.99	.007
Cruciferous	0.97	0.95, 0.99	.027	0.99	0.97, 1.02	.751
Tomatoes	1.00	0.99, 1.01	.636	1.00	0.99, 1.01	.342
Green vegetables	1.02	1.00, 1.03	.033	1.01	0.99, 1.03	.508
Onions	1.01	1.00, 102	.043	1.00	0.98, 1.02	.930
Other vegetables	1.00	0.99, 1.01	.140	1.00	0.99, 1.01	.358
Salt	1.31	1.01, 1.70	.040	1.30	0.90, 1.88	.164
Fruits on trees	1.00	1.00, 1.01	<.001	1.00	1.00, 1.01	.049
Citrus fruits	1.00	0.99, 1.01	.145	1.01	0.99, 1.01	.289
Fruits on ground	1.01	0.99, 1.02	.062	1.01	0.99, 1.02	.323
Dried fruits	1.00	0.99, 1.01	.987	1.00	0.99, 1.02	.665
Fruit juice	1.01	1.00, 1.01	.015	1.01	0.99, 1.01	.076
Hydrogenated fats	1.01	0.99, 1.02	.194	1.02	1.00, 1.03	.018
Liquid oils (UFAs)	1.02	0.97, 1.07	.524	0.99	0.92, 1.06	.732
Olives	1.17	1.09, 1.26	<.001	1.15	1.07, 1.25	<.001
Nuts	1.05	1.02, 1.08	.002	1.02	0.98, 1.07	.290

Abbreviations: CD, Crohn's disease; RR, relative risk; UC, ulcerative colitis; UFAs: unsaturated fatty acids.

^a^ Wald test in multinominal logistic regression analysis for the association between food groups intakes (continuous and independent) and IBD categories (categorical and dependent), adjusted for age, sex, education, job, marriage, smoke, BMI, and physical activity; the healthy group was considered as a the reference group (relative risk ratio 1.00).

**TABLE 6:** Logistic regression analysis between IBD categories and food groups intake (*N* = 234)

Food groups intake	IBD^a^		
	Odds ratio	95% CI	*p* value^b^
Whole grains	1.00	0.99, 1.00	.055
Refined grains	0.99	0.993, 0.998	.001
Potato	0.96	0.94, 0.98	<.001
Sweets	0.99	0.97, 1.00	.062
Sugars	0.99	0.97, 1.01	.166
Honey and jam	1.05	1.00, 1.10	.032
Snacks	0.98	0.95, 0.99	.036
Soft drinks	0.99	0.99, 1.00	.307
Tea	1.00	0.99, 1.00	.612
Coffee	1.00	0.99, 1.00	.125
Legumes	0.90	0.85, 0.94	<.001
Red meat	0.99	0.98, 1.01	.399
Poultry meat	1.01	0.97, 1.06	.595
Sea food	1.04	1.02, 1.07	.002
Processed meat	0.96	0.91, 1.01	.090
Organ meat	1.10	1.04, 1.16	.001
Eggs	1.01	0.99, 1.02	.447
Fast foods	0.98	0.95, 1.00	.106
Low‐fat dairy	0.99	0.99, 1.00	.565
High‐fat dairy	0.98	0.98, 0.99	<.001
Cruciferous	0.98	0.96, 0.99	.042
Tomatoes	1.00	0.99, 1.01	.461
Green vegetables	1.01	1.00, 1.03	.048
Onions	1.01	0.99, 1.02	.086
Other vegetables	1.00	0.99, 1.01	.125
Salt	1.30	1.02, 1.65	.036
Fruits on trees	1.00	1.00, 1.01	<.001
Citrus fruits	1.01	0.99, 1.01	.119
Fruits on ground	1.01	0.99, 1.02	.073
Dried fruits	1.00	0.99, 1.01	.826
Fruit juice	1.01	1.00, 1.01	.012
Hydrogenated fats	1.01	0.99, 1.02	.058
Liquid oil (UFAs)	1.01	0.96, 1.06	.684
Olives	1.17	1.10, 1.25	<.001
Nuts	1.04	1.01, 1.07	.003

Abbreviations: IBD, inflammatory bowel diseases, UFAs, unsaturated fatty acids.

^a^ IBD ulcerative (colitis and Crohn's disease).

^b^ Wald test in logistic regression analysis for the association between food groups intake (continuous, independent) and IBD (binary and dependent), adjusted for age, sex, education, job, marriage, smoke, BMI, and physical activity; the healthy group was considered as a reference group, (Odds ratio 1.00).

## 4 DISCUSSION

The current study examined the association between macro‐ and several micronutrient intake and food groups with the risk of IBD and its subgroups (UC and CD). The main results displayed that the higher amount of protein intake derived from seafood and cholesterol was related to an increased risk of IBD and UC development, while patients who had a higher intake of calcium were less likely to have CD compared to healthy individuals. Regarding food groups, we found a notably direct association between the ingestion of honey and jam, seafood, organ meats, salt, fruits on trees, fruit juice, olives, and nuts and the probability of IBD and UC development; meanwhile, a significant inverse association was detected for refined grains, potatoes, salty snacks, legumes, high‐fat dairy, and cruciferous in comparison to healthy subjects. Additionally, individuals, who had a higher intake of organ meats, fruits on trees, hydrogenated fats, and olives were more likely to have CD, while participants with a higher intake of refined grains, sweets, salty snacks, legumes, and high‐fat dairy products were less probable to have CD.

Our findings were in agreement with two prospective cohort studies; in this field, it has demonstrated a straight relation between the consumption of animal proteins and not plant proteins with the increased risk of UC (Vidal‐Lletjós et al., 2017). Interestingly, among the different sources of animal proteins, consumption of a greater amount of meat or fish but not eggs or dairy products has been associated with IBD risk (Jantchou et al., 2010; Shoda et al., 1996). In contrast, Hart and coworkers found no significant association between diet and UC risk (Hart et al., 2008).

Inconsistent with our reports, a case–control study conducted on 62 UC patients from Iran observed no relationship between total protein, bean, nut, fish, poultry, egg, and dairy products and UC risk (Rezazadeh et al., 2020). The different results might be associated with different sample sizes, different study designs, and populations. Existing evidence confirms a potential association between diet and the composition and activity of the gut microbiota (Flint et al., 2008; Vidal‐Lletjós et al., 2017). However, it remains still largely unclear regarding the role of dietary composition in terms of macronutrients, vitamins, and minerals on the intestinal mucosa. Importantly, it should be noticed that the nutritional effect of diet could be varied among CD and UC patients (Vidal‐Lletjós et al., 2017).

Our study found that participants who had a substantially higher calcium consumption were less likely to have CD compared to healthy controls. Consistent with our findings, a cross‐sectional study in kids with IBD indicated that calcium intake differed significantly in kids with CD (Sila et al., 2019). Inadequate calcium intake is seen in 80%–86% of IBD patients through several reasons such as malabsorption, lactose intolerance, and lower consumption of milk and dairy products (Kilby et al., 2019). IBD patients have lower serum calcium and phosphate levels than healthy individuals (Ratajczak et al., 2020). Due to the lack of calcium intake, supplementation with this mineral can have positive effects on IBD patients. The positive effect of calcium supplementation in IBD patients may be related to modification of gut microbiomes such as *Bifidobacterium*, *Akkermansia*, and *Ruminococcus* (Yang et al., 2020).

According to the available data, it has also been revealed that there exists a significant relationship between the higher intake of whole grains, honey and jam, seafoods, organ meats, green vegetables, onions, salt, fruits on trees, fruit juice, olives, and nuts and a higher risk for developing UC, whereas the higher intake of refined grains, potatoes, legumes, high‐fat dairy products, and cruciferous was inversely linked with UC incidence versus healthy subjects.

Besides, a remarkable direct trend was elucidated between the consumption of organ meats, fruits on trees, hydrogenated fats, and olives and increased risk for CD, while a substantial link was identified between higher intake of refined grains, sweets, salty snacks, legumes, and high‐fat dairy products and lower the incidence of CD.

A narrative review regarding the effects of food groups in IBD patients presented that high protein intake, especially animal protein containing organic sulfur such as fish or meat rather than dairy products or eggs consumption, was associated with a significant increase in the risk of IBD (Campmans‐Kuijpers & Dijkstra, 2021). Our results were the same as that study.

In contrast, Barnes et al showed that there was no significant relationship between odds of disease relapse among UC patients and intake of meat, especially those containing sulfur (Barnes et al., 2017). The inconsistent findings might be related to the different study designs. Barnes' study was designed to assess the risk of relapse in a known case of UC but our study had been designed to assess the risk of IBD development.

According to Campmans‐Kuijpers et al review study, whole grains are less likely to be consumed in CD patients and UC and CD patients were also more likely to consume refined grains. It seems that IBD patients consume a FODMAP diet (a diet low in fermentable oligosaccharides, disaccharides, monosaccharides, and polyols) to reduce gastrointestinal symptoms. The results of the present study showed that consumption of refined grains was inversely linked with the incidence of both UC and CD and the higher intake of whole grains was directly linked with the incidence of UC (Ratajczak et al., 2020). Despite positive results from consuming whole grains and its macroscopic and microscopic lesion improvements in animal studies, a cohort study did not show a significant association between consumption of whole grains and dietary fiber and prevention of UC (Andersen et al., 2018; Prasadi et al., 2020).

Another study found that potato and legume consumption was inversely associated with IBD relapse (Ratajczak et al., 2020).

The report of the current study demonstrated that a higher intake of honey and jam is related to a higher incidence of UC, and a higher intake of sweets is related to a lower incidence of CD. Analysis of the nutrients and food groups intake of Polish males with UC showed that sugar consumption in male patients with UC was significantly higher than in the control group, but there were no significant differences between honey and jam consumption among the two groups (Głąbska et al., 2019). One study found that restriction of simple sugars other than honey has beneficial effects in IBD patients. Beneficial effects of honey consumption have been seen in patients with CD (Ratajczak et al., 2020).

In our study, higher salt intake was associated with an increased risk of UC, and surprisingly, salty snack consumption was associated with a reduced risk of CD. The narrative review indicated that the data on salt consumption and its relationship with IBD are contradictory.

Contrary to our study, consumption of fruit is associated with a reduced risk of CD and UC. However, there are differences in the types of fruits, for example, grapefruit consumption usually increased symptoms in IBD patients. Besides, our observations indicated that a significant inverse association was detected between consumption of cruciferous vegetables and UC/ IBD incidence. Similarly, the recent review illustrated cruciferous vegetables containing indole‐3‐carbinol, which activates the aryl hydrocarbon receptor (AhR), had a beneficial effect on IBD (Campmans‐Kuijpers & Dijkstra, 2021). AhR is a transcription factor that plays a protective role in IBD. AhR can suppress COX‐2 expression (Wang et al., 2018).

Our observations in this study illustrated a relationship between the consumption of high‐fat dairy products and a lower incidence rate of CD. The systematic review and meta‐analysis indicated that increased consumption of high‐fat milk products was positively associated with the risk of UC and CD. One reason for the conflicting results in this systematic review might be related to the consumption of high‐fat dairy products that is popular in western dietary patter and it might be just one of the factors in developing IBD (Li et al., 2020).

Our research had some strengths such as this was a case–control study on treatment‐naïve IBD patients for the first time in Iran, with the various dietary intakes and patterns owing to different cultures and socioeconomic statuses. Allocation between case and control groups for confounding factors such as age, sex, and BMI was another important strength. However, this study had some limitations: first of all, FFQ instrument has a limited number of food items; and self‐reported questionnaire and recall bias were inevitable and unsolved. It seems that combination of different methods, such as the FFQ with dietary record (or 24‐hour recall) and/or the FFQ along with nutritional biomarkers might be more accurate than each method separately for dietary intake assessment. Additionally, selection bias owing to its case–control design is another important weakness. However, our researchers carefully selected the studied participants as controls and cases. Indeed, the obtained findings were restricted to one population and may not be representative of the overall general population.

## 5 CONCLUSIONS

In brief, we found that higher protein from seafoods and cholesterol intake was positively associated with a higher risk of IBD development. A significant association between honey and jam, seafoods, organ meats, salt, fruits on trees, fruit juice, olives, and nuts consumption and IBD development was found in this study. A large‐sample, case–control study with a comprehensive daily report food questionnaire should be suggested.

## CONFLICT OF INTEREST

The authors declare that they do not have any conflict of interest.

## ETHICAL STATEMENT

The study was approved by the Research Vice Chancellor of Iran University of Medical Sciences (No: IR.IUMS.REC139631962).

## INFORMED CONSENT

After a complete explanation, informed written consent was obtained from all the participants.

## Supporting information


Table S1

## Data Availability

The datasets used during the current study are available from the corresponding author, Marjan Mokhtare, on reasonable request.
