# Supplementary material for: Evaluating macro‐ and micronutrients and food groups intake with the risk of developing inflammatory bowel disease: Is there any association?
Source: Food Sci Nutr. 2022 Jul 26;10(11):3920–30. doi: 10.1002/fsn3.2988 (PMC9632195; doi:10.1002/fsn3.2988)
Supplement: Supplementary file 1 — Table S1 [file FSN3-10-3920-s001.docx]

**Supplementary Table 1**. Food groups (n=36) and their food items used in principal component analysis to derive dietary patterns

| **Food categories** | **Food groups (used in PCA)** | **Food items included** |
| --- | --- | --- |
| Cereal (2) | Whole grains | Whole grain bread, rice with bran, |
|  | Refined grains | White bread, White rice, Pasta, noodles, flour, biscuits |
| Legumes (1) | Legumes | Lentils, beans, chickpeas, split chickpeas, canned legumes and mixed legumes |
|  |  |  |
| Vegetables (7 groups) | Potatoes | Red and white potato, baked potato, sweet potato, peeled potato, boiled potato |
|  | Green vegetables | Spinach, coriander, parsley, fenugreek, grape leaves, tarragon, lettuce, cabbage (red and white) |
|  | Dried vegetables | Spinach, leek, coriander, parsley, basil and fenugreek |
|  | Tomato | Raw, peeled and boiled tomatoes, tomato paste |
|  | Onions | Raw, peeled onion, fried onion, boiled onion |
|  | Other vegetables | Carrot, beetroot, garlic, leek, radish |
|  | Cruciferous | Cabbage, broccoli |
| Fruits (5 groups) | Citrus fruits | Orange, tangerine, grapefruit, lemon |
|  | Fruits grown on ground | Melons, watermelons |
|  | Fruits grown on trees | Plum, pomegranate, fig, apple, mango, grapes, cherries, apricot, and canned fruits |
|  | Fresh fruit juice | Grape juice, apple juice, orange juice, peach juice, mango juice |
|  | Dried fruits | Dried apricot, peach, |
| Meat and alternative  (7 groups) | Red meat | lamb, beef, mixed meat and organ meat |
|  | Poultry meat | Chicken, other poultry meat and canned poultry meat |
|  | Processed meat | Sausages and salami |
|  | Organ meat | Liver, heart, brain, intestine |
|  | Fish and seafood | Fish, shrimp, other sea food and tuna |
|  | Eggs | All types of eggs |
|  | Nuts | Peanut, pistachio, almond, walnut |
| Dairy products (2 groups) | Low fat dairy products | Skimmed and low fat <3 % fat milk, yoghurt, and curd |
|  | High fat dairy products | High fat >3% milk and yoghurt, all types of cheese, ice cream, |
| Fats and oils (3 groups) | Hydrogenated fats | Hydrogenated fats (solid fats), cream |
|  | Liquid oils | Oils such as walnut oil , sunflower oil |
|  | Olives | Olives, olive liquid |
| Confectionary (4 groups) | Sugars | Table sugars, hard sugars (cubes) |
|  | Sweets | Candies, chocolates, cakes |
|  | Snacks | Chips and cheese puffs |
|  | Honey and jam | Honey, jam |
| Beverages (3 groups) | Tea | Tea |
|  | Coffee | Coffee |
|  | Soft drinks | Coca cola, Pepsi, Seven up, other carbonated drinks |
| Fast-foods (1 group) | Fast foods | Hamburgers, pizza |
| Salt (1 group) | Salt | Salt at table and cooking |
